# Deplatforming did not decrease Parler users’ activity on fringe social media

**DOI:** 10.1093/pnasnexus/pgad035

**Published:** 2023-03-21

**Authors:** Manoel Horta Ribeiro, Homa Hosseinmardi, Robert West, Duncan J Watts

**Affiliations:** School of Computer and Communication Sciences, EPFL, 1015 Lausanne, Philadelphia, Switzerland; Computational Social Science Lab, University of Pennsylvania, PA 19104, USA; School of Computer and Communication Sciences, EPFL, 1015 Lausanne, Philadelphia, Switzerland; Computational Social Science Lab, University of Pennsylvania, PA 19104, USA

**Keywords:** deplatforming, content moderation, social networks, social media‌

## Abstract

Online platforms have banned (“deplatformed”) influencers, communities, and even entire websites to reduce content deemed harmful. Deplatformed users often migrate to alternative platforms, which raises concerns about the effectiveness of deplatforming. Here, we study the deplatforming of Parler, a fringe social media platform, between 2021 January 11 and 2021 February 25, in the aftermath of the US Capitol riot. Using two large panels that capture longitudinal user-level activity across mainstream and fringe social media content (*N* = 112, 705, adjusted to be representative of US desktop and mobile users), we find that other fringe social media, such as Gab and Rumble, prospered after Parler’s deplatforming. Further, the overall activity on fringe social media increased while Parler was offline. Using a difference-in-differences analysis (*N* = 996), we then identify the causal effect of deplatforming on active Parler users, finding that deplatforming increased the probability of daily activity across other fringe social media in early 2021 by 10.9 percentage points (pp) (95% CI [5.9 pp, 15.9 pp]) on desktop devices, and by 15.9 pp (95% CI [10.2 pp, 21.7 pp]) on mobile devices, without decreasing activity on fringe social media in general (including Parler). Our results indicate that the isolated deplatforming of a major fringe platform was ineffective at reducing overall user activity on fringe social media.

**Significance Statement**

Deplatforming is a common practice among online platforms to reduce content deemed harmful. However, its effectiveness has been debated, as impacted users, influencers, or communities often migrate to alternative platforms. Using two large panels capturing the activity of US mobile and desktop users across mainstream and fringe social media, we study the deplatforming of Parler, a social media platform associated with conspiracy theorists and far-right extremists. Our results indicate that deplatforming a major fringe platform in isolation was ineffective at reducing overall user activity on fringe social media, as users migrated to alternate platforms like Gab or Rumble.

## Introduction

To address the rise of false information, hateful speech, and conspiracy theories, online platforms have banned influencers, communities, and even entire websites associated with content deemed harmful (1). Such actions, broadly referred to as “deplatforming,” often result in the migration of affected users to other, more permissive platforms (2). Because individual platforms typically lack access to other platforms’ data, the overall effectiveness of deplatforming is hard to evaluate conclusively. Reflecting this difficulty, previous work has focused on the effects of deplatforming users and communities within a single platform (3) or between a pair of platforms where one was the object of enforcement and the other was set up explicitly as a substitute (4, 5). In general, these studies have found that deplatforming leads to a decrease in overall harmfulness, albeit an increase in the harmfulness of users who remain active (3–5). The designs of existing analyses have, however, two important limitations. First, they do not consider the full range of websites users might migrate to after deplatforming, especially less public-facing platforms such as Telegram (6). Second, they rely on active engagement (e.g. tweeting), which may underestimate the passive consumption of harmful content (e.g. views that do not lead to tweets). These limitations make it hard to obtain comprehensive and reliable estimates of the effects of deplatforming.

In this paper, we address these limitations in the context of a high-profile deplatforming event: the suspension of the US social networking service Parler from Amazon’s Web hosting services on 2021 January 11, following the US Capitol attack. At the time, Parler was associated with conspiracy theorists and far-right extremists (7) and had around 2.3 million daily active users (1). During the shutdown, Parler users were reported to have migrated to other fringe social media such as Rumble, Gab, and Telegram. To capture the total consumption of fringe content across various social media platforms (see *Materials and methods* for a complete list), we analyzed two large panels from the Nielsen Company encompassing US desktop (*N*_Desktop_ = 76, 677) and mobile (*N*_Mobile_ = 36, 028) users from August 2020 to June 2021 (adjusted to be representative of US desktop and mobile users). These data's user-level, longitudinal nature allows us to identify the causal effect of deplatforming on Parler users. In sum, we find that Parler’s temporary removal effectively stopped on-platform activity but caused a surge in activity on other fringe sites that roughly compensated for the drop-off, resulting in a negligible overall effect.

## Results

**Fig. 1.**
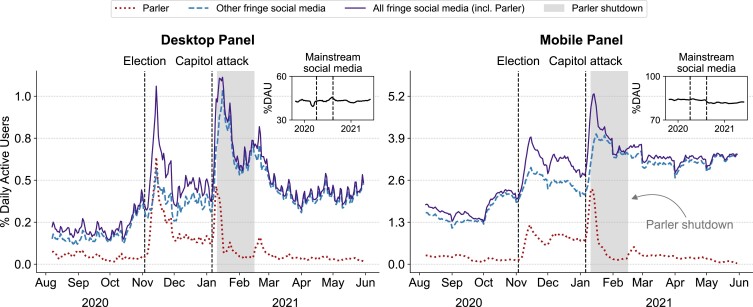
Daily percentage of US desktop (left) and mobile (right) users who were active on Parler (dotted line), other fringe social media websites (dashed line), and across all fringe social media (including Parler; solid line), smoothed with a 7-day moving average. Users were weighted so that the panel was representative of the US population (see Supplementary Material, Appendix). Insets show user activity on mainstream social media sites (e.g. Facebook, Twitter, and YouTube) for comparison. Parler’s number of active users is non-zero during the shutdown period because some users still navigate toward the defunct domain. After the deplatforming of Parler, other fringe platforms (e.g. Rumble, Gab) prospered and the overall consumption of fringe social media increased.

Fig. 1 shows the estimated percentage of daily active users (i.e. who visited the websites or mobile apps) of Parler and other fringe social media considering US desktop (left panel) and mobile (right) users (see cf. Supplementary Material, Appendix). User activity on Parler and other fringe social media grew sharply in the aftermath of the 2020 US Presidential Election and peaked following the US Capitol attack on 2021 January 6. For example, Parler’s percentage of daily active users among all panelists increased by more than six times from its highest value in October (desktop: 0.12%; mobile: 0.25%; Fig. 1 is smoothed by a 7-day moving average) and the week following the election (desktop: 0.83%; mobile: 1.54%), and increased again after January 6 (desktop: 0.96%; mobile: 3.5%). On January 11, Parler was deplatformed by Amazon, leading to a decrease in daily active users through February 25, when Parler was relaunched on another hosting service (daily activity was not precisely zero post-shutdown as some users still navigated to the defunct domain). Over this same period, however, the percentage of daily active users of fringe social media platforms other than Parler surged both on desktop and mobile to such an extent that user activity across all fringe platforms was higher during the shutdown period than before. By comparison, activity on mainstream social media (e.g. Facebook) remained roughly constant over this interval (see Fig. 1 insets).

**Fig. 2.**
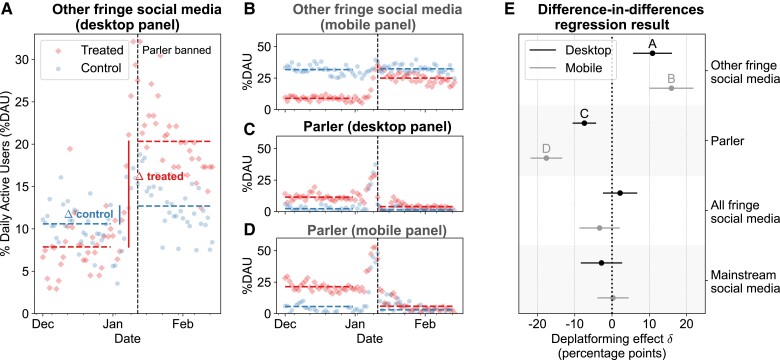
(A–D) Considering treated users (active on Parler pre-deplatforming; diamond marker) and control users (active on other fringe social media but not Parler, pre-deplatforming; circle marker), we show the percentage of daily active users across different types of platforms for the desktop and mobile panels between 2020 December 1 and 2021 February 25. (A) illustrates the difference-in-differences model (DiD). (E) DiD regression results for the desktop (black) and mobile (gray) panels. Coefficients from scenarios depicted in (A–D) are annotated with the corresponding letters in (E). Error bars represent 95% confidence intervals. The DiD coefficients indicate that deplatforming increased the probability of daily activity across other fringe social media and did not significantly decrease activity on fringe social media in general.

This last result seems to suggest that deplatforming Parler backfired, causing users to migrate to other venues with similar (fringe) content. However, the increase could also have been driven by non-Parler users whose behavior was affected by other factors (e.g. increased media attention to fringe content around January 6). In other words, aggregate data of the sort shown in Fig. 1 cannot identify the causal effect of deplatforming on Parler users. To overcome this hurdle, we apply a difference-in-differences (DiD) approach to longitudinal user-level activity in the desktop and mobile panels. We consider the month of December 2020 as our pre-intervention period and the days between 2021 January 11 and 2021 February 25 as the post-intervention period. Our DiD approach compares two matched groups of panelists (cf. *Materials and methods*), illustrated in Fig. 2A: “treated” users who spent over 3 min on Parler in December 2020 ($N_{\text{Desktop}}^{\text{Treated}}=135$; $N_{\text{Mobile}}^{\text{Treated}}=209$) and “control” users who spent over 3 min on other fringe social media platforms and less than 3 min on Parler over the same period ($N_{\text{Desktop}}^{\text{Control}}=265$; $N_{\text{Mobile}}^{\text{Control}}=387$). Our model calculates the difference in probability of daily activity (i.e. the chance of a user visiting a website or set of websites on a given day) between the pre- and post-intervention periods for both treatment and control groups (Δtreated and Δcontrol in Fig. 2A). Under the identifying assumption that these differences would remain constant in the absence of the intervention (here, the deplatforming of Parler), we can estimate its causal effect through the difference in differences (*δ* = Δtreated − Δcontrol; cf. *Materials and methods* for details). Fig. 2A–D shows the same information for all four combinations of Parler vs. other fringe social media and desktop vs. mobile, where parallel pre-intervention trends across all four panels suggest that the identifying assumption is credible (as does placebo testing; see Supplementary Material, Appendix).

Fig. 2E depicts the results of the DiD regression analysis (cf. *Materials and methods*). We observe a significant decrease in the probability of daily activity on Parler itself for both the desktop (−7.4 percentage points; 95% CI [ − 10.4, − 4.4]) and mobile (−17.6; 95% CI [ − 21.7, − 13.5]) panels. Consistent with Fig. 1, however, there was also a significant increase in the time spent by active Parler users on *other* fringe social media for both panels (desktop: 10.9 percentage points; 95% CI [5.8, 15.9]; mobile: 15.9; 95% CI [10.2, 21.7]). In sum, the net effect of deplatforming on Parler users over *all* fringe social media (Parler as well as others) was small and not statistically significant. As a sanity check, we run the same model comparing the user activity on mainstream social media websites (e.g. YouTube, Twitter, cf. Supplementary Material, Appendix for a complete list), which we would not expect to be affected by the deplatforming, again finding small and statistically insignificant effects.

## Discussion

These results indicate that deplatforming Parler was ineffective at reducing the consumption of the type of content that was deplatformed. It *increased* user activity on other fringe social media platforms and did not significantly decrease the total user activity on all fringe social media platforms taken together. This finding is aligned with previous research suggesting that online hate groups are resilient to uncoordinated interventions that affect only part of their ecosystem (8). Web stakeholders may benefit from this insight by reconsidering platform-level interventions and by, e.g. promoting simultaneous action against multiple fringe social media platforms or acting proactively rather than reactively during periods of political unrest. Our analysis is also relevant to researchers studying content moderation in general, as it demonstrates the value of analyzing passive engagement across multiple platforms. Measurements capturing overt engagement (e.g. posts, comments) on specific platforms (3) or pairs of platforms (5, 4) tend to underestimate user activity on fringe social media after deplatforming, as users move to a variety of other platforms (some, like Telegram, less public-facing).

Our findings are naturally limited to the deplatforming of an entire social media (Parler) during a period of exceptional political unrest. We note, however, that our scenario is similar to other instances of deplatforming; e.g. 8kun, and Gab, here analyzed as part of the “other fringe social media” category, were themselves temporarily deplatformed after being associated with mass shootings (9). Deplatforming policies applied to individual actors (e.g. Twitter users, YouTube channel owners) or groups (e.g. subreddits) may have different effects from those observed here. We also note that the validity of our causal results is predicated on the identifying assumptions of our DiD model.

Fringe platforms are an important part of an ecosystem of fringe communities and personalities who exert influence over the media (2, 10) and large mainstream social media (11). They are tied to hate crimes (9) and anti-democratic riots (12), and were pivotal to the so-called “infodemic” during the 2020 coronavirus pandemic (13). In this context, we hope our findings will help inform policy responses to such websites.

## Materials and methods

### Data

Our data are drawn from panels maintained by the Nielsen Company, where individuals agree to have their media and Internet consumption habits tracked in exchange for payment. Specifically, we use two such panels: a desktop panel and a mobile panel. The panels have rotating membership, meaning that tracked individuals join and leave the panel over time. In both panels, tracking software is installed on the user’s devices (their computer for the desktop panel and their phone for the mobile panel).

### Labeling social media platforms

To analyze user activity on the fringe and mainstream social media across the panels, we create lists of apps and domains and then detect panelists accessing websites/apps in the list. We consider fringe social media platforms to be broadly what Freelon et al. (2) define as “Alt-tech” platforms: social media websites that have become popular among groups espousing extreme or fringe opinions due to moderation policies that are less stringent than those of mainstream social media such as Facebook or YouTube. Specifically, we consider domains and apps associated with Locals, Gab, Rumble, BitChute, 8kun, Telegram, 4chan, MeWe, DLive, Minds, and Parler. We consider mainstream social media platforms to be those included in Pew’s 2021 survey about social media use (14).

### Difference-in-differences

We estimate the effect *δ* of deplatforming Parler on users’ social media usage with a DiD model:


(1) $$Y_{it}=\gamma P_{t}+\lambda T_{i}+\delta P_{t}T_{i}+\epsilon_{it},$$

where the daily usage *Y*_*it*_ of user *i* on day *t* is determined by whether day *t* came after the deplatforming of Parler (*P*_*t*_ ∈ {0, 1}) and whether the user was an active consumer of Parler before the intervention (*T*_*i*_ ∈ {0, 1}).

We make our results more robust by estimating our DiD model using weights generated by coarsened exact matching and clustering standard errors at the user level. We matched users on sociodemographic characteristics and their pre-intervention activity (using Scott binning). We achieved exact matches for the sociodemographic features and low standardized mean differences for pre-intervention activity (0.028 for desktop; 0.011 for mobile). To obtain such matching, we discard 346 units in the mobile panel (out of 942) and 112 units in the desktop panel (out of 512), obtaining matched samples of size *N*_Desktop_ = 400 and *N*_Mobile_ = 596.

## Supplementary Material

pgad035_Supplementary_Data

## Data Availability

We are unable to share data for this paper. We have been exempted from PNAS Nexus Materials and Data Availability Policy by the editorial board upon request.
