# Correction to: Deplatforming did not decrease Parler users’ activity on fringe social media

**DOI:** 10.1093/pnasnexus/pgad159

**Published:** 2023-05-18

This is a correction to: Manoel Horta Ribeiro, Homa Hosseinmardi, Robert West, Duncan J Watts, Deplatforming did not decrease Parler users’ activity on fringe social media, *PNAS Nexus*, Volume 2, Issue 3, March 2023, pgad035, https://doi.org/10.1093/pnasnexus/pgad035

In the originally published PDF version of this manuscript, the second paragraph of the abstract read “Here, we study the deplatforming of Parler, a fringe social media platform, between 2020 January 11 and 2021 February 25…” This should have said “Here, we study the deplatforming of Parler, a fringe social media platform, between 2021 January 11 and 2021 February 25…”

This error has been corrected.

