# Supplementary material for: Deplatforming did not decrease Parler users’ activity on fringe social media
Source: PNAS Nexus. 2023 Mar 21;2(3):pgad035. doi: 10.1093/pnasnexus/pgad035 (PMC10029837; doi:10.1093/pnasnexus/pgad035)
Supplement: pgad035_Supplementary_Data [file pgad035_supplementary_data.pdf]

Supplementary Information for

Deplatforming did not decrease Parler users' activity on fringe social media

Manoel Horta Ribeiro, Homa Hosseinmardi, Robert West, Duncan J. Watts

Data Science Lab, EPFL, 1015 Lausanne, Switzerland

Computational Social Science Lab, University of Pennsylvania, Philadelphia, PA 19104, United States

To whom correspondence should be addressed. E-mail: manoel.hortaribeiro@epfl.ch

This PDF file includes:

Supplementary text
SI References

Supporting Information Text

Extended methods: Data

Desktop panel description. In the desktop panel, users are asked to install tracking software on their web browser(s). When the users open their browser and access a website, a "session" is initiated. A session is finished on four occasions: When the URL is changed, the tab is deactivated, the browser is closed, or the computer ceases to be in "awake" mode. A single row of the desktop panel data corresponds to a session. A session contains the timestamp of when the session started, the URL associated with the session, and the duration (the time spent between the beginning and the end of the session). Our desktop panel (N = 76,677) had the following sociodemographic characteristics:

- Gender – 62.8% of participants were women.
- Race/Ethnicity – 17.7% of participants were Black, 3.8% of participants were Asian or Pacific Islanders, 1.3% of participants were American Indian or Alaska natives, 12.9% of participants were Hispanic.
- Education – 28.7% of participants had higher school-level education or lower, 41.1% of participants attended some college, 20.3% of participants were college graduates, 9.9% of participants completed post-graduate degrees.
- Income – 23.9% of participants' households earned less than 25 thousand US dollars per year, 28.4% earned between 25 and 50 thousand US dollars per year, 19.8% earned between 50 and 75 thousand US dollars per year, 12.5% earned between 75 and 100 thousand US dollars per year, 15.3% earned more than 100 thousand US dollars per year.
- Age — 6.2% of participants were between 18 and 20 years old, 43.4% of participants were between 21 and 44 years old, 31.5% of participants were between 45 and 64 years old, 13.9% of participants were 65 years old or older.

Mobile panel description. In the mobile panel, users are asked to install a tracking app on their phones. The app runs in the background and records users' Web history and app usage. Like the desktop panel, Nielsen then divides users' phone usage into sessions, each corresponding to a single row of the mobile panel data. However, sessions related to app usage (e.g., opening the Mail app) and Web browsing (i.e., opening a Web browser and surfing on the Web) are different: Web browsing sessions contain the domain of the URL visited, the timestamp of when the session started, and the duration of the session. App usage sessions contain the app's name (e.g., the Mail app), the starting timestamp, and the session duration. Our desktop panel (N = 36,028) had the following sociodemographic characteristics:

- Gender – 56.6% of participants were women.
- Race/Ethnicity – 18.3% of participants were Black, 3.5% of participants were Asian or Pacific Islanders, 1.7% of participants were American Indian or Alaska natives, 21.3% of participants were Hispanic.
- Education – 29.7% of participants had higher school-level education or lower, 42.5% of participants attended some college, 18.7% of participants were college graduates, 9.0% of participants completed post-graduate degrees.
- Income – 0.8% of participants' households earned less than 25 thousand US dollars per year, 29.4% earned between 25 and 50 thousand US dollars per year, 17.5% earned between 50 and 75 thousand US dollars per year, 10.4% earned between 75 and 100 thousand US dollars per year, 12.0% earned more than 100 thousand US dollars per year.
- Age — 5.9% of participants were between 18 and 20 years old, 50.9% of participants were between 21 and 44 years old, 35.2% of participants were between 45 and 64 years old, 8.1% of participants were 65 years old or older.

Data used to study platform-level trends. When analyzing the overall user activity across Parler and other fringe social media, we consider the entire panel between August 2020 and June 2021. In total, there were N = 76,677 unique participants in the desktop panel and N = 36,028 unique participants in the mobile panel. Across the considered time span, the average time in the panel was 5.5 months for desktop and 4.5 months for mobile. The Nielsen Company also provides weights accompanying each panel; each individual i is assigned a weight w_i such that the weights map to the US population ($\sum w_i \approx$ number of individuals in the US using desktop/mobile). We use these weights for the analysis done in Fig. 1 of the paper when analyzing the percentage of daily active users (%DAU). We define the %DAU as the percentage of people in the panel that accessed a social media platform (e.g., Parler) or a category of social media platforms (e.g., mainstream) on a given day. If on day t the %DAU for Parler is 1%, this means that per 100 panelists enrolled on day t, 1 had a session where Parler was accessed. Note that while we calculate this percentage, we employ the demographic weights provided by the Nielsen Company to adjust our sample.

Data used to study the user-level impact of deplatforming. When analyzing the effect of deplatforming on active users on Parler, we considered two sets of matched users, namely (1) those who spent over 3 minutes browsing Parler in December 2020 ($N^{\text{Treated}}_{\text{Desktop}} = 135$; $N^{\text{Treated}}_{\text{Mobile}} = 209$), termed "treated"; and (2) those who spent over 3 minutes browsing other fringe social networking platforms and less than 3 minutes on Parler over the same period ($N^{\text{Control}}_{\text{Desktop}} = 265$; $N^{\text{Control}}_{\text{Mobile}} = 387$), termed "control". The outcome we consider for our user-level analysis is the daily activity (1 if a user visited the domain/app associated with a social media platform at least once on the respective day, and 0 otherwise).

Further information about panels. Panelists receive up to $60 in rewards points per year and participate in monthly $10,000 sweepstakes that are spread over 400 winners (top prize earners win $1,000). The panels rely on convenience recruitment with demographic targets. According to Nielsen, panels under-represent males, persons 18–24 and older than 65 years old, as well as incomes under $75,000 per year. The skew is corrected by weighting to the universe of smartphone and tablet owners, separated by the operating system (for the mobile panel) and to the universe of individuals with access to a desktop computer at home or work (for the desktop panel). For the mobile panel, age, gender, race, Hispanic ethnicity, and income are used as controls. For the desktop panel, controls are gender, age, education, household size, income, Hispanic ethnicity, working status, designated metropolitan area, and Spanish-language dominance. There may be users in both panels, although we do not have this information at hand. Yet, we do not foresee this will impact the results obtained. In contrast, we argue that having analyses on two different panels (even if they share panelists) strengthens our findings.

List of mainstream social media considered. YouTube, Facebook, Instagram, Pinterest, LinkedIn, Snapchat, Twitter, WhatsApp, TikTok, Reddit, Nextdoor.

Extended methods: Difference-in-differences

Difference-in-differences approach. To estimate the effect δ of the deplatforming of Parler on users' social media usage, we use a difference-in-differences (DiD) model:

$$Y_{it} = \gamma P_t + \lambda T_i + \delta P_t T_i + \epsilon_{it}, \qquad [1]$$

where the daily usage Y_{it} of user i on day t is determined by whether the day t came after the deplatforming of Parler (P_t) and whether the user was an active consumer of Parler before the intervention (T_i). With this specification, we estimate the coefficient δ associated with the interaction between the dummy variables P_t and T_i using OLS to obtain the average treatment effect on the treated (ATT). Given the parallel trends assumption, we have that $\hat{\delta}$ is an estimate of the effect of being active on Parler ($T_i = 1$) on the usage metric after the intervention ($P_t = 1$):

$$\hat{\delta} = E[Y_{ij} \mid P_t = 1, T_i = 1] - E[Y_{ij} \mid P_t = 1, T_i = 0]. \qquad [2]$$

Following Yang et al. (1), we make our results more robust by estimating our DiD model using weights generated by coarsened exact matching (2) (CEM) and cluster standard errors at the level of the user i [since the daily activity time-series of each user may be autocorrelated, see (3)]. Our ability to causally identify ATTs with the described DiD strategy is predicated on a key identifying assumption: in the absence of the deplatforming event (the "treatment"), the difference between users that were active on Parler and those that were not (treated and control groups) remains constant over time for the considered outcomes. Time series of outcomes of different matched samples shown in Fig. 2 of the paper suggest that this assumption is plausible, as the time series for treated and control groups appear to move in parallel before the deplatforming of Parler. This assumption is relaxed due to our usage of coarsened exact matching, as the CEM-based results are valid as long as any differences in how the two groups would have evolved in the absence/presence of the intervention are entirely explained by the panelist characteristics on which we match.

Coarsened exact matching. To perform coarsened exact matching, we assign each panelist to a stratum based on their age, race, ethnicity, gender, education level, income, and pre-intervention level of consumption of fringe social media [binned using Scott's normal reference rule (4)]. For each panelist i in a stratum s containing a mixture of panelists that were and were not active Parler users before deplatforming ("treated" vs. "untreated"), we construct a CEM weight

$$w_i = \begin{cases} 1 & \text{if } T_i = 1, \\ \frac{N_{T=0}}{N_{T=1}} \frac{N^s_{T=1}}{N^s_{T=0}} & \text{if } T_i = 0, \end{cases} \qquad [3]$$

where $N_{T=1}$ ($N_{T=0}$) is the total number of treated (untreated) panelists and $N^s_{T=1}$ ($N^s_{T=0}$) is the number of treated (untreated) panelists in stratum s. We perform this matching with the R package *MatchIt* (5).

Placebo testing. To test the robustness of the parallel trends assumption, we carry out a placebo test. We use the same control and treatment groups as before and the same difference-in-differences models specified in Eq. 1. However, we consider the first 15 days of December as the pre-treatment period (i.e., $P_t = 0$ for t between December 1 and 15, 2020) and the last 16 days of December as the post-treatment period (i.e., $P_t = 1$ for t between December 16 and 31, 2020). Since there was no intervention on December 15, the parallel trends assumption here implies that there should be no significant difference between treatment and control groups. This is indeed what we find: all coefficients obtained are small (smaller than 2.5 percentage points) and not statistically significant ($p > 0.05$).

References

1. L Yang, et al., The effects of remote work on collaboration among information workers. *Nat. human behaviour* **6**, 43–54 (2022).
2. SM Iacus, G King, G Porro, Causal inference without balance checking: Coarsened exact matching. *Polit. analysis* **20**, 1–24 (2012).
3. M Bertrand, E Duflo, S Mullainathan, How much should we trust differences-in-differences estimates? *The Q. journal economics* **119**, 249–275 (2004).
4. DW Scott, On optimal and data-based histograms. *Biometrika* **66**, 605–610 (1979).
5. EA Stuart, G King, K Imai, D Ho, Matchit: nonparametric preprocessing for parametric causal inference. *J. statistical software* (2011).
